# Genetic variations in methotrexate metabolic pathway genes influence methotrexate responses in rheumatoid arthritis patients in Malaysia

**DOI:** 10.1038/s41598-022-15991-0

**Published:** 2022-07-13

**Authors:** Hong Xi Sha, Kumar Veerapen, Sook Khuan Chow, Suk Chyn Gun, Ing Soo Lau, Renee Lay Hong Lim, Zaliha Zulkifli, Yoon-Yen Yow, Suat Cheng Peh, Jung Shan Hwang

**Affiliations:** 1 Department of Biological Sciences, School of Medical and Life Sciences, Sunway University, Bandar Sunway, Selangor Darul Ehsan Malaysia; 2 Analytic and Translational Genetics Unit, Massachusetts General Hospital, Boston, MA USA; 3 Sunway Medical Centre, Bandar Sunway, Selangor Darul Ehsan Malaysia; 4 Department of Medicine, Hospital Tuanku Ja’afar Seremban, Seremban, Negeri Sembilan Malaysia; 5 Department of Medicine, Hospital Selayang, Batu Caves, Selangor Darul Ehsan Malaysia; 6 Department of Biotechnology, Faculty of Applied Sciences, UCSI University, Cheras, Kuala Lumpur, Malaysia; 7 Department of Medical Sciences, School of Medical and Life Sciences, Sunway University, 47500 Bandar Sunway, Selangor Darul Ehsan Malaysia; 8 Stanley Center for Psychiatric Genetics, Broad Institute of MIT and Harvard, Cambridge, MA USA

**Keywords:** Drug discovery, Genetics, Medical research, Rheumatology, Risk factors

## Abstract

Methotrexate (MTX) is the most widely used disease-modifying anti-rheumatic drug (DMARD) for rheumatoid arthritis (RA). Many studies have attempted to understand the genetic risk factors that affect the therapeutic outcomes in RA patients treated with MTX. Unlike other studies that focus on the populations of Caucasians, Indian and east Asian countries, this study investigated the impacts of six single nucleotide polymorphisms (SNPs) that are hypothesized to affect the outcomes of MTX treatment in Malaysian RA patients. A total of 647 RA patients from three ethnicities (N_Malay_ = 153; N_Chinese_ = 326; N_Indian_ = 168) who received MTX monotherapy (minimum 15 mg per week) were sampled from three hospitals in Malaysia. SNPs were genotyped in patients using TaqMan real-time PCR assay. Data obtained were statistically analysed for the association between SNPs and MTX efficacy and toxicity. Analysis of all 647 RA patients indicated that none of the SNPs has influence on either MTX efficacy or MTX toxicity according to the Chi-square test and binary logistic regression. However, stratification by self-identified ancestries revealed that two out of six SNPs, *ATIC* C347G (rs2372536) (OR 0.5478, 95% CI 0.3396–0.8835, p = 0.01321) and *ATIC* T675C (rs4673993) (OR 0.5247, 95% CI 0.3248–0.8478, p = 0.008111), were significantly associated with MTX adequate response in RA patients with Malay ancestry (p < 0.05). As for the MTX toxicity, no significant association was identified for any SNPs selected in this study. Taken all together, *ATIC* C347G and *ATIC* T675C can be further evaluated on their impact in MTX efficacy using larger ancestry-specific cohort, and also incorporating high-order gene–gene and gene–environment interactions.

## Introduction

Rheumatoid arthritis (RA) is an autoimmune disorder which abnormally attacks normal joints and results in inflammation. Most epidemiological studies of RA have been done in Western countries, showing a prevalence of RA in the range of 0.5–1.0% in the USA and northern European countries^1^. In Malaysia, 0.5% of the population is affected by RA^2^. It should be noted that there are 3 main ancestries in Malaysia, being 69.8% Malays, 22.4% Chinese and 6.8% Indian as according to the 2021 press release by the Department of Statistics Malaysia^3^. Currently, the most commonly prescribed medication for RA is disease-modifying anti-rheumatoid drugs (DMARDs) including conventional synthetic DMARDs (csDMARDS), targeted synthetic DMARDs (tsDMARDS) and biologic DMARDs (bDMARDs). Other prescribed drugs include non-steroidal anti-inflammatory drugs (NSAIDs) and glucocorticoids^4^. Previous csDMARDs such as anti-malarial drugs (chloroquine and hydroxychloroquine) have been in use since the 1950s^5^. Since methotrexate (MTX) was readapted in the late 1980s, it has become the most widely used csDMARD^6^.

MTX is a folate anti-metabolite that suppresses disease activity and reduces joint pain. A low dose (15–25 mg) of MTX per week is prescribed to patients through either subcutaneous or oral administration for at least three months and this drug has been proven to be an effective DMARD for RA^7,8^. However, about 30–50% of RA patients do not respond to MTX and thus ruling out MTX as a treatment option for these non-responders^9–13^. Moreover, up to 35% RA patients are forced to discontinue MTX due to the adverse drug effects including stomatitis, gastrointestinal upset, headache or minor central nervous system disturbance, hair loss, ulcers, liver toxicity, pancytopenia and pneumonitis^6,9–13^. Despite these limitations, MTX is still the gold standard for the treatment of RA and in Malaysia, the usage of MTX has increased sixfold from 1997 to 2007^14^. Malaysia Clinical Practice Guidelines on RA recommends that patients are to take a combination of MTX with non-biologic DMARDs or folic acid (minimum 5 mg/week) when MTX monotherapy shows signs of failure in efficacy or side effects, respectively^15^. Hence, anti-RA medicine can become unexpectedly lengthy, costly and ranges from not effective to partially effective in meeting treatment expectation.

Available literature has converged to hypothesize that the variability in MTX efficacy and toxicity is due to a dysregulation in the MTX pathway (Supplementary Fig. S1)^16–18^. As multiple enzymes mediate the metabolism of MTX, it is conceivable that the alterations of enzymes' availability and activity have a direct impact on MTX treatment. Our study focused on the potential dysregulation of 4 key enzymes: folylpolyglutamate synthase (FPGS), γ-glutamyl hydrolase (GGH), aminoimidazole-4-carboxamide ribonucleotide (AICAR) transformylase, and inosine triphosphate pyrophosphatase (ITPA). FPGS is an important enzyme responsible for converting MTX into a range of polyglutamate forms—methotrexate polyglutamate (MTX PG) and genetic variants of FPGS likely affect the retention of MTX bioavailable in the cell. The therapeutic effects of MTX in RA patients rely on its conversion to MTX PG^18^. The conversion of MTX to MTX PG by FPGS can be reversed by GGH^16^ (Supplementary Fig. S1). GGH removes the polyglutamates from MTX PG by performing serial trimming on the long chain MTX PG to yield MTX which is able to be exported from the cell. Therefore, variants of *GGH* such as rs11545078 (C452T) causes defective GGH and leads to an increase of intracellular concentration of MTX PG^19^, rs3758149 (C-401 T), on the other hand, promotes enzymatic activity of GGH that results in the hydrolysis of MTX PG^20^. AICAR transformylase (encoded by *ATIC* gene) converts AICAR to formyl-AICAR (FAICAR), playing a role in the de novo purine synthesis (Supplementary Fig. S1). MTX is able to block AICAR transformylase and hence causes an increase of intracellular level of AICAR which has a role in the activation of adenosine signaling pathway, resulting in a series of anti-inflammatory activities^21^. Several studies shown two variants of *ATIC*, C347G (rs2372536)^20,22^ and T675C (rs4673993)^23,24^ achieve good clinical response to MTX. ITPA is also played an important role in the adenosine signaling pathway. The presence of C94A (rs1127354; chr20:3213196) mutation may modulate the ability of ITPA to convert inosine triphosphate (ITP) to inosine monophosphate (IMP) required for de novo purine synthesis which will affect the amount of intracellular adenosines which can be exported for binding to adenosine receptors^21^, leading to multiple anti-inflammatory mechanisms.

Since FPGS, GGH, AICAR and ITPA are known to determine the rate limiting steps in MTX metabolism, the effects of genetic variations such as single nucleotide polymorphisms (SNPs) could modulate the pharmacokinetic properties of these enzymes^17,23,25–27^. In our study, six SNPs were selected for the genotyping analysis as based on their previously reported association with MTX treatment outcomes. These SNPs include *FPGS* A1994G (rs10106; chr9:127813796), *GGH* C452T (rs11545078; chr8:63026205), *GGH* C401T (rs3758149; chr8:63039169), *ATIC* C347G (rs2372536; chr2:215325297), *ATIC* T675C (rs4673993; chr2:215347616) and *ITPA* C94A (rs1127354; chr20:3213196). In addition, these candidate gene association studies were previously and mainly conducted in the Caucasian population and no similar study has been carried out for Malaysian RA patients. Therefore, the novelty of this study is to reassess the possible association of the SNPs with MTX treatment outcome in a Malaysian population. We hypothesize that specific SNP changes that can alter gene function are able to explain the variability observed in MTX efficacy and toxicity. Thus, this study aimed to genotype six SNPs of the candidate genes (*FPGS*, *GGH*, *ATIC* and *ITPA*) in MTX metabolic pathway and determine their association with MTX therapeutic outcomes in Malaysian RA patients.

## Results

### Characterization of the studied population

*Demographics*. This study recruited 647 RA patients from Sunway Medical Centre (n = 268), Hospital Tuanku Ja'afar Seremban (n = 284) and Hospital Selayang (n = 95) (Table 1). The number of Chinese RA patients, most of which were recruited from Sunway Medical Centre, was twice the amount compared to the number of Malay or Indian RA patients. This account for the skewed distribution of the ancestry-specific groups in our patient sampling with over 80% of patients recruited from the private hospital (Sunway Medical Centre) were Chinese (N = 326), compared to the number for Malay (N = 153) or Indian (N = 168) patients. Shahrir et al.^28^ was the first to report on the number of RA registries in Malaysia, showed majority of RA patients in Malaysia are Indian (54.5%), followed by Malay (31.4%), Chinese (11.6%), Indigenous (1.2%) and others (1.3%). However, the authors also admitted the possibility of incomplete data because they have not included RA patients from private and university hospitals in their study. In 2008, the population sizes of Malay, Chinese and Indian were 53.3%, 26.0% and 7.7%, respectively. Due to the lack of information of the prevalence of RA in different ancestry-specific groups in Malaysia, and due to limitation in the sampling of patients, we cannot conclusively determine the prevalence of RA in the three main ancestry-specific groups of Malays, Chinese and Indians in Malaysia. The female RA patients (88.7%) outnumbered male RA patients (11.3%) in this study. This sex-imbalance is consistent with the current literature which have shown a higher number of female RA patients.

**Table 1:** Characteristics of the patients enrolled in this study.

Characteristics	Total^a^	Malay	Chinese	Indian
Patients number, n (%)	647(100%)	153 (23.65%)	326 (50.39%)	168 (25.97%)
**Demographics**				
**Gender**				
Female, n (%)	574 (88.72%)	131 (85.62%)	289 (88.65%)	154 (91.67%)
Male, n (%)	73 (11.28%)	22 (14.38%)	37 (11.35%)	14 (8.33%)
**Age (years)**				
Mean (SD)	56 (12.10)	53 (11.63)	58 (12.02)	56 (11.95)
Range	18–92	18–83	21–92	18–92
**Age of disease diagnosis (years)**				
Mean (SD)	46 (12.46)	44 (12.39)	48 (12.51)	46 (12.15)
Range	9–89	11–80	9–80	16–89
**Disease duration (years)**				
Mean (SD)	10 (7.39)	9 (6.98)	10 (7.67)	10 (7.12)
Range	0.5–47	1–36	0.5–46	1–47
**Clinical data**				
**RF**^**b**^				
RF positive RA, n (%)	525 (81.14%)	121 (79.08%)	262 (80.37%)	142 (84.52%)
RF negative RA, n (%)	121 (18.70%)	32 (20.92%)	63 (19.33%)	26 (15.48%)
**Anti-CCP**^**c**^				
Anti-CCP positive RA, n (%)	490 (75.73%)	108 (70.59%)	259 (79.45%)	123 (73.21%)
Anti-CCP negative RA, n (%)	128 (19.78%)	43 (28.10%)	52 (15.95%)	33 (19.64%)
**MTX efficacy, n (%)**				
AR^d^	252 (41.79%)	59 (41.55%)	129 (42.16%)	64 (41.03%)
IR^e^	352 (58.21%)	83 (58.45%)	177 (57.84%)	92 (58.97%)
**MTX toxicity, n (%)**				
Non-ADR^f^	448 (69.24%)	104 (67.97%)	236 (72.39%)	109 (64.88%)
ADR^g^	199 (30.76%)	49 (32.03%)	90 (27.61%)	59 (35.12%)

A total of 647 RA patients were stratified into three ancestry-specific groups, Malay, Chinese and Indian.

^a^Data are presented in number (percentage) or mean (standard deviation) unless otherwise indicated; ^b^Rheumatoid factor; ^c^Anti-cyclic citrullinated peptide; ^d^Adequate responder; ^e^Inadequate responder; ^f^Non-adverse drug reaction; ^g^Adverse drug reaction.

#### MTX efficacy and toxicity

Based on our criteria for categorization for MTX efficacy, we obtained a total of 252 adequate responders (ARs) and 352 inadequate responders (IRs): 58% of RA patients did not respond well to MTX (Table 1). As for MTX toxicity, we identified 448 non-adverse drug reaction (Non-ADR) and 199 adverse drug reaction (ADR) patients: 1 in 3 RA patients developed at least one type of side effects during the MTX treatment. Among 199 RA patients who experienced ADRs, 43 patients showed severe side effects and their MTX therapy were immediately ceased (Table 1). These 43 patients were excluded from the MTX efficacy analysis but included in the MTX toxicity analysis.

### Differences of allelic and genotype frequencies among 3 ancestry-specific groups

The TaqMan SNP genotyping assay was performed on all study samples for the following SNPs: *FPGS* A1994G (rs10106), *GGH* C452T (rs11545078), *GGH* C401T (rs3758149), *ATIC* C347G (rs2372536), *ATIC* T675C (rs4673993), and *ITPA* C94A (rs1127354). 5% of the samples were randomly chosen for each SNPs and then verified by Sanger sequencing. The sequencing results confirmed the accuarry of the TaqMan SNP genotyping assay results. The allelic frequencies and genotype counts for each SNPs in Malay, Chinese and Indian RA patients are shown in Table 2. The minor allele frequency (MAF) of all six SNPs, except *ITPA* C94A (rs1127354), showed significant variation among the RA patients for the three ancestry-specific groups. The MAFs of *ITPA* C94A (rs1127354) in Malay, Chinese and Indian patients are 0.15, 0.16 and 0.14, respectively (p > 0.05; solid-line box in Table 2). In addition, genotype counts for the six SNPs were compared among the three ancestry-specific groups using chi-square test. The results revealed that except *GGH* C452T (rs11545078) and *ITPA* C94A (rs1127354), other four SNPs significantly differ in genotype frequencies among the Malay, Chinese and Indian RA patients (dashed line boxes in Table 2).

**Table 2:** Allele and genotype frequencies of the three ancestry-specific groups in Malaysia.

SNP			Total	Malay	Chinese	Indian
*FPGS* A1994G (rs10106)	Genotype Count^a^	AA	111 (17.16%)	21 (13.73%)	36 (11.04%)	54 (32.14%)
		AG	283 (43.74%)	74 (48.37%)	129 (39.57%)	80 (47.62%)
		GG	253 (39.10%)	58 (37.91%)	161 (49.39%)	34 (20.24%)
	Minor allele frequency^b^		0.61 (789)	0.62 (190)****	0.69 (451)****	0.44 (148)****
*GGH* C452T (rs11545078)	Genotype Count^a^	CC	521 (80.53%)	117 (76.47%)	276 (84.66%)	128 (76.19%)
		CT	116 (17.93%)	33 (21.57%)	48 (14.72%)	35 (20.83%)
		TT	10 (1.55%)	3 (1.96%)	2 (0.61%)	5 (2.98%)
	Minor allele frequency^b^		0.11 (136)	0.13 (39)*	0.08 (52)*	0.13 (45)*
*GGH* C401T (rs3758149)	Genotype Count^a^	CC	356 (55.02%)	69 (45.10%)	198 (60.74%)	89 (52.98%)
		CT	237 (36.63%)	63 (41.18%)	112 (34.36%)	62 (36.90%)
		TT	54 (8.35%)	21 (13.73%)	16 (4.91%)	17 (10.12%)
	Minor allele frequency^b^		0.27 (345)	0.34 (105)***	0.22 (144)***	0.29 (96)***
*ATIC* C347G (rs2372536)	Genotype Count^a^	CC	239 (36.94%)	44 (28.76%)	158 (48.47%)	37 (22.02%)
		CG	292 (45.13%)	80 (52.29%)	133 (40.80%)	79 (47.02%)
		GG	116 (17.93%)	29 (18.95%)	35 (10.74%)	52 (30.95%)
	Minor allele frequency^b^		0.40 (524)	0.45 (138)****	0.31 (203)****	0.54 (183)****
*ATIC* T675C (rs4673993)	Genotype Count^a^	TT	241 (37.25%)	46 (30.07%)	159 (48.77%)	36 (21.43%)
		TC	296 (45.75%)	80 (52.29%)	135 (41.41%)	81 (48.21%)
		CC	110 (17.00%)	27 (17.65%)	32 (9.82%)	51 (30.36%)
	Minor allele frequency^b^		0.40 (516)	0.44 (134)****	0.31 (199)****	0.54 (183)****
*ITPA* C94A(rs1127354)	Genotype Count^a^	CC	460 (71.10%)	110 (71.90%)	226 (69.33%)	124 (73.81%)
		CA	177 (18.08%)	40 (26.14%)	96 (29.45%)	41 (24.40%)
		AA	10 (1.55%)	3 (1.96%)	4 (1.23%)	3 (1.79%)
	Minor allele frequency^b^		0.15 (197)	0.15 (46)	0.16 (104)	0.14 (47)

*P ≤ 0.05; **P ≤ 0.01; ***P ≤ 0.001; ****P ≤ 0.0001.

^a^Data are presented in number (percentage) unless otherwise indicated. ^b^Data are presented in frequency (number) unless otherwise indicated.

### Association of six metabolic SNPs with MTX efficacy and toxicity in three ancestry-specific RA patients

**Figure 1:**
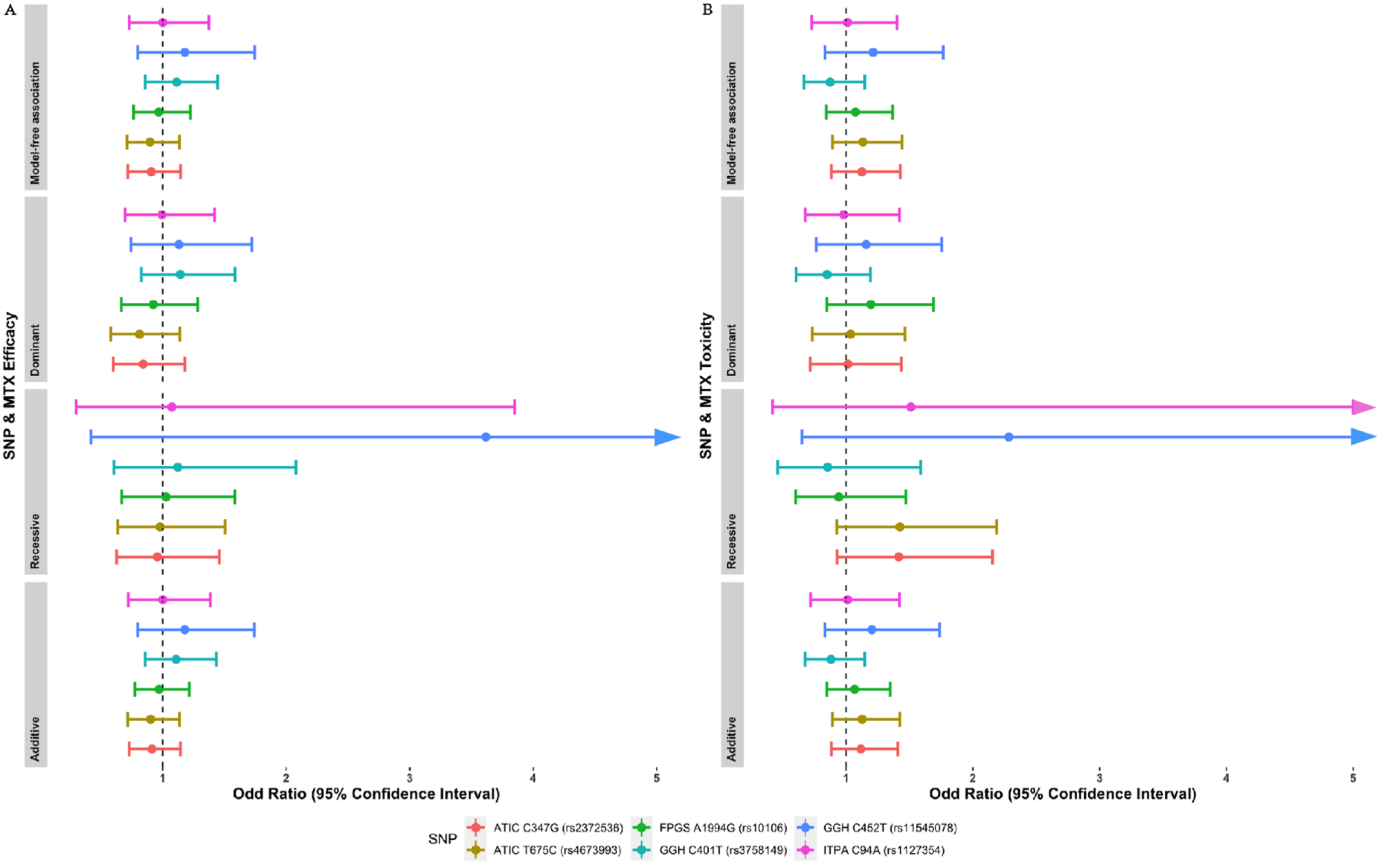
Forest plot showing the association between SNPs and either MTX efficacy or MTX Toxicity. (**A**) Forest plot showing the association between SNPs and MTX efficacy; (**B**) Forest plot showing the association between SNPs and MTX toxicity. A logarithmic scale was applied on the x-axis. Circle points represent the OR of each test performed, and the results of 95% CI were displayed as a horizontal line. All the tests crossed the vertical line (OR 1.0), indicating that no significant association was found.

When the association study of SNPs with the MTX treatment was carried out using the entire cohort (n = 647), there was no significant difference between ARs and IRs as well as Non-ADR groups and ADR groups in the allelic association tests. Logistic regression was then performed to test the standard models of disease penetrance (dominant, recessive, additive) for the interaction of six SNPs with MTX efficacy and toxicity in the cohort of 647 RA patients. The forest plot (Fig. 1) for the association between SNPs and the MTX efficacy and toxicity was performed using R package ggplot2^29,30^ indicated no significant association between the SNPs with either MTX efficacy or MTX toxicity (Table 3).

**Table 3:** The association between SNPs in enzymes mediating MTX metabolism and MTX efficacy and toxicity.

SNP		MTX efficacy						MTX toxicity					
		AR^a^	IR^b^	Model	OR^c^	95% CI^d^	p-value^e^	Non-ADR^a^	ADR^b^	Model	OR^c^	95% CI^d^	p-value^e^
*FPGS* A1994G (rs10106)	AA	42 (41.18%)^g^	60 (58.82%)^g^	Dominant	0.9237	0.664–1.285	0.6376	78 (70.27%)^g^	33 (29.73%)^g^	Dominant	1.196	0.8466–1.689	0.3102
	AG	111 (43.02%)	147 (56.98%)	Recessive	1.027	0.6668–1.583	0.1225	189 (66.78%)	94 (33.22%)	Recessive	0.943	0.6035–1.473	0.7966
	GG	99 (40.74%)	144 (59.26%)	Additive	0.9709	0.7755–1.216	0.7968	181 (71.83%)	71 (28.17%)	Additive	1.068	0.8467–1.347	0.5787
	MAF^f^	0.61	0.62	Model-free	0.9682	0.7653–1.225	0.7875	0.61	0.60	Model-free	1.074	0.8434–1.367	0.5637
*GGH* C452T (rs11545078)	CC	208 (42.36%)	283 (57.64%)	Dominant	1.132	0.7442–1.721	0.5625	364 (70.00%)	156 (30.00%)	Dominant	1.159	0.7655–1.755	0.4853
	CT	43 (40.57%)	63 (59.43%)	Recessive	3.617	0.42–31.15	1.17	79 (68.10%)	37 (31.90%)	Recessive	2.284	0.6536–7.978	0.1958
	TT	1 (16.67%)	5 (83.33%)	Additive	1.179	0.7985–1.742	0.4071	5 (50.00%)	5 (50.00%)	Additive	1.204	0.8342–1.737	0.3218
	MAF	0.09	0.10	Model-free	1.18	0.7986–1.744	0.4056	0.10	0.12	Model-free	1.214	0.8345–1.767	0.3099
*GGH* C401T (rs3758149)	CC	143 (43.33%)	187 (56.67%)	Dominant	1.144	0.8265–1.585	0.4166	241 (67.89%)	114 (32.11%)	Dominant	0.8504	0.6071–1.191	0.3461
	CT	91 (40.09%)	136 (59.91%)	Recessive	1.123	0.6071–2.079	0.3707	168 (70.89%)	69 (29.11%)	Recessive	0.8549	0.4597–1.59	0.6205
	TT	18 (39.13%)	28 (60.87%)	Additive	1.109	0.8583–1.434	0.4277	39 (72.22%)	15 (27.78%)	Additive	0.8817	0.678–1.147	0.3477
	MAF	0.25	0.27	Model-free	1.113	0.8578–1.445	0.42	0.27	0.25	Model-free	0.8749	0.6675–1.147	0.3326
*ATIC* C347G (rs2372536)	CC	88 (39.29%)	136 (60.71%)	Dominant	0.8421	0.6017–1.179	0.3163	166 (69.75%)	72 (30.25%)	Dominant	1.016	0.7187–1.436	0.9283
	CG	118 (43.54%)	153 (56.46%)	Recessive	0.9574	0.6284–1.459	− 0.2025	209 (71.58%)	83 (28.42%)	Recessive	1.416	0.9299–2.156	0.105
	GG	46 (42.59%)	62 (57.41%)	Additive	0.9137	0.7293–1.145	0.4329	73 (62.93%)	43 (37.07%)	Additive	1.117	0.8851–1.409	0.3516
	MAF	0.42	0.39	Model-free	0.9082	0.7195–1.146	0.4176	0.40	0.43	Model-free	1.125	0.885–1.429	0.3365
*ATIC* T675C (rs4673993)	TT	88 (38.60%)	140 (61.40%)	Dominant	0.8125	0.5809–1.137	0.2254	168 (69.71%)	73 (30.29%)	Dominant	1.036	0.7327–1.464	0.8428
	TC	121 (44.32%)	152 (55.68%)	Recessive	0.9787	0.6359–1.506	− 0.09773	211 (71.53%)	84 (28.47%)	Recessive	1.425	0.9284–2.188	0.1052
	CC	43 (42.16%)	59 (57.84%)	Additive	0.9027	0.7187–1.134	0.3786	69 (62.73%)	41 (37.27%)	Additive	1.127	0.8909–1.426	0.3188
	MAF	0.41	0.3 s	Model-free	0.898	0.711–1.134	0.3664	0.39	0.42	Model-free	1.133	0.8912–1.441	0.3077
*ITPA* C94A (rs1127354)	CC	178 (41.78%)	248 (58.22%)	Dominant	0.995	0.6975–1.419	0.978	318 (69.28%)	141 (30.72%)	Dominant	0.9819	0.679–1.42	0.9227
	CA	70 (41.92%)	97 (58.08%)	Recessive	1.075	0.3002–3.85	0.1113	124 (70.06%)	53 (29.94%)	Recessive	1.511	0.4217–5.415	0.5261
	AA	4 (40.00%)	6 (60.00%)	Additive	1.001	0.7225–1.386	0.9974	6 (60.00%)	4 (40.00%)	Additive	1.012	0.7216–1.42	0.9437
	MAF	0.15	0.16	Model-free	1.001	0.7293–1.373	0.9974	0.15	0.15	Model-free	1.012	0.7286–1.404	0.9455

^a^Adequate responder; ^b^Inadequate responder; ^c^OR: odd ratio; ^d^95% CI: 95% confidence intervals frequency; ^e^*p*-value: probability value, considering a statistically significant two-sided probability (p) value less than 0.05; ^f^MAF: Minor allele frequency; ^g^Data are presented in number (percentage) unless otherwise indicated.

**Figure 2:**
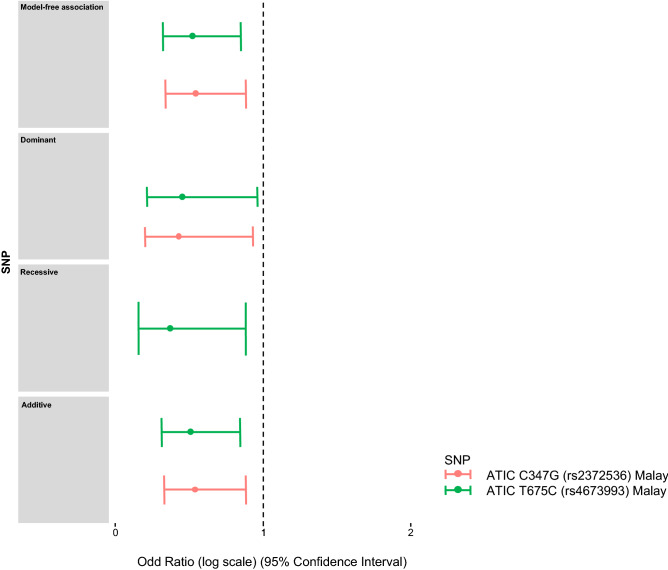
Forest plot showing a significant correlation of two *ATIC* SNPs with MTX efficacy in Malay RA patients. The forest plot was plotted by a logarithmic scale on the x-axis. The cycle dot represents the OR of each test performed, and the results of 95% CI were displayed as a horizontal line.

We then stratified the study cohort into three ancestry-specific groups, Malay, Chinese and Indian. The numbers of AR and IR of the stratified groups with their respective genotypes are presented in Table 4. Using these data, significant differences were observed in *ATIC* C347G (rs2372536) (OR 0.5478, 95% CI 0.3396–0.8835, p = 0.01321) and *ATIC* T675C (rs4673993) (OR 0.5247, 95% CI 0.3248–0.8478, p = 0.008111) (Table 5). Based on the effect sizes obtained from our analyses, the risk of patients becoming IR was reduced by *ATIC* C347G (rs2372536) and *ATIC* T675C (rs4673993) for approximately 55% and 57%, respectively. When the inheritance models were applied to the ancestry-specific stratification, it could be inferred that: (i) *ATIC* C347G (rs2372536) was associated with AR in Malay RA patients under dominant and additive models; (ii) the minor allele of *ATIC* T675C (rs4673993) under three genetic models (dominant, recessive and additive) may predict a higher success rate in MTX treatment among Malay RA patients (Table 5). All positive results for the association between SNPs and MTX efficacy are as shown in the forest plot (Fig. 2). All the six SNPs were not significantly associated with the MTX efficacy in either Chinese or Indian RA patients. Furthermore, there was no significant association of all six SNPs with MTX toxicity in the three ancestry-specific groups (Tables 4, 6). It is worth mentioning a recent paper concluded an association of MTX side effects with *FPGS* A1994G (rs10106) of the south Indian Tamil with RA^31^. More than 80% of Malaysian Indian study subjects are also of Tamil origin, despite this the association between MTX toxicity and the *FPGS* A1994G (rs10106) in this ancestry-specific group is not evident in our study.

**Table 4:** Genotype and minor allele frequencies in three different ethnic groups in response to MTX efficacy and toxicity.

Ethnic group		MTX efficacy						MTX toxicity						
		Malay		Chinese		Indian		Malay		Chinese			Indian	
Gene variant		AR^a^	IR^b^	AR	IR	AR	IR	Non-ADR^c^	ADR^d^	Non-ADR	ADR		Non-ADR	ADR
*FPGS* A1994G (rs10106)	AA	7 (35.00%)^e^	13 (65.00%)	17 (51.52%)	16 (48.48%)	18 (36.73%)	31 (63.27%)	16 (76.19%)^c^	5 (23.81%)	25 (69.44%)	11 (30.56%)		37 (68.52%)	17 (31.48%)
	AG	33 (50.77%)	32 (49.23%)	44 (36.97%)	75 (63.03%)	34 (45.95%)	40 (54.05%)	48 (64.86%)	26 (35.14%)	92 (71.32%)	37 (28.68%)		49 (61.25%)	31 (38.75%)
	GG	19 (33.33%)	38 (66.67%)	68 (44.16%)	86 (55.84%)	12 (36.36%)	21 (63.64%)	40 (68.97%)	18 (31.03%)	118 (73.29%)	43 (26.71%)		23 (67.65%)	11 (32.35%)
	MAF^f^	0.60	0.65	0.70	0.70	0.45	0.45	0.62	0.63	0.70	0.68		0.44	0.45
*GGH* C452T (rs11545078)	CC	42 (38.89%)	66 (61.11%)	115 (43.73%)	148 (56.27%)	51 (42.15%)	70 (57.85%)	77 (65.81%)	40 (34.19%)	201 (72.83%)	75 (27.17%)		86 (67.19%)	42 (32.81%)
	CT	16 (50.00%)	16 (50.00%)	14 (34.15%)	27 (65.85%)	13 (39.39%)	20 (60.61%)	25 (75.76%)	8 (24.24%)	32 (66.67%)	16 (33.33%)		22 (62.86%)	13 (37.14%)
	TT	1 (50.00%)	1 (50.00%)	0 (0.00%)	2 (100.00%)	0 (0.00%)	2 (100.00%)	2 (66.67%)	1 (33.33%)	2 (100.00%)	0 (0.00%)		1 (20.00%)	4 (80.00%)
	MAF	0.15	0.11	0.05	0.09	0.10	0.13	0.14	0.10	0.08	0.09		0.11	0.18
*GGH* C401T (rs3758149)	CC	26 (41.94%)	36 (58.06%)	84 (45.16%)	102 (54.84%)	33 (39.76%)	50 (60.24%)	45 (65.22%)	24 (34.78%)	140 (70.71%)	58 (29.29%)		56 (62.92%)	33 (37.08%)
	CT	22 (36.67%)	38 (63.33%)	42 (39.62%)	64 (60.38%)	27 (44.26%)	34 (55.74%)	41 (65.08%)	22 (34.92%)	83 (74.11%)	29 (25.89%)		44 (70.97%)	18 (29.03%)
	TT	11 (55.00%)	9 (45.00%)	3 (21.43%)	11 (78.57%)	4 (33.33%)	8 (66.67%)	18 (85.71%)	3 (14.29%)	12 (75.00%)	4 (25.00%)		9 (52.94%)	8 (47.06%)
	MAF	0.37	0.34	0.19	0.24	0.27	0.27	0.37	0.29	0.23	0.20		0.28	0.29
*GGH* C16T (rs1800909)	CC	11 (55.00%)	9 (45.00%)	3 (21.43%)	11 (78.57%)	4 (33.33%)	8 (66.67%)	18 (85.71%)	3 (14.29%)	12 (75.00%)	4 (25.00%)		9 (52.94%)	8 (47.06%)
	CT	22 (36.67%)	38 (63.33%)	43 (40.95%)	62 (59.05%)	27 (43.55%)	35 (56.45%)	41 (65.08%)	22 (34.92%)	82 (73.87%)	29 (26.13%)		44 (69.84%)	19 (30.16%)
	TT	26 (41.94%)	36 (58.06%)	83 (44.39%)	104 (55.61%)	33 (40.24%)	49 (59.76%)	45 (65.22%)	24 (34.78%)	141 (70.85%)	58 (29.15%)		56 (63.64%)	32 (36.36%)
	MAF	0.63	0.66	0.81	0.79	0.73	0.72	0.63	0.71	0.77	0.80		0.72	0.70
*ATIC* C347G (rs2372536)	CC	12 (27.91%)	31 (72.09%)	59 (40.14%)	88 (59.86%)	17 (48.57%)	18 (51.43%)	30 (68.18%)	14 (31.82%)	114 (72.15%)	44 (27.85%)		22 (59.46%)	15 (40.54%)
	CG	31 (43.66%)	40 (56.34%)	59 (46.83%)	67 (53.17%)	28 (37.84%)	46 (62.16%)	54 (67.50%)	26 (32.50%)	98 (73.68%)	35 (26.32%)		57 (72.15%)	22 (27.85%)
	GG	16 (57.14%)	12 (42.86%)	11 (33.33%)	22 (66.67%)	19 (40.43%)	28 (59.57%)	20 (68.97%)	9 (31.03%)	23 (65.71%)	12 (34.29%)		30 (57.69%)	22 (42.31%)
	MAF	0.53	0.39	0.31	0.31	0.52	0.55	0.45	0.45	0.31	0.32		0.54	0.56
*ATIC* T675C (rs4673993)	TT	13 (28.89%)	32 (71.11%)	59 (39.86%)	89 (60.14%)	16 (45.71%)	19 (54.29%)	32 (69.57%)	14 (30.43%)	114 (71.70%)	45 (28.30%)		22 (61.11%)	14 (38.89%)
	TC	30 (42.25%)	41 (57.75%)	61 (47.66%)	67 (52.34%)	30 (40.00%)	45 (60.00%)	53 (66.25%)	27 (33.75%)	100 (74.07%)	35 (25.93%)		58 (71.60%)	23 (28.40%)
	CC	16 (61.54%)	10 (38.46%)	9 (30.00%)	21 (70.00%)	18 (39.13%)	28 (60.87%)	19 (70.37%)	8 (29.63%)	21 (65.63%)	11 (34.38%)		29 (56.86%)	22 (43.14%)
	MAF	0.53	0.39	0.31	0.31	0.52	0.55	0.44	0.44	0.30	0.31		0.53	0.57
*ITPA* C94A (rs1127354)	CC	42 (41.18%)	60 (58.82%)	90 (42.45%)	122 (57.55%)	46 (40.71%)	67 (59.29%)	75 (68.18%)	35 (31.82%)	164 (72.57%)	62 (27.43%)		79 (63.71%)	45 (36.29%)
	CA	15 (40.54%)	22 (59.46%)	38 (42.22%)	52 (57.78%)	17 (42.50%)	23 (57.50%)	28 (70.00%)	12 (30.00%)	67 (69.79%)	29 (30.21%)		29 (70.73%)	12 (29.27%)
	AA	2 (66.67%)	1 (33.33%)	1 (25.00%)	3 (75.00%)	1 (33.33%)	2 (66.67%)	1 (33.33%)	2 (66.67%)	4 (100.00%)	0 (0.00%)		1 (33.33%)	2 (66.67%)
	MAF	0.16	0.14	0.16	0.16	0.15	0.15	0.14	0.16	0.16	0.16		0.14	0.14

^a^Adequate responder; ^b^Inadequate responder; ^c^Non-adverse drug reaction; ^d^Adverse drug reaction. ^e^Data are presented in number (percentage) unless otherwise indicated; ^f^MAF: Minor allele frequency.

**Table 5:** The asscoacition between SNPs and MTX efficacy in three different ancestry-specific groups.

SNP	Model	Malay			Chinese			Indian		
		OR^a^	95% CI^b^	p-value^c^	OR	95% CI	p-value	OR	95% CI	p-value
*FPGS* A1994G (rs10106)	Dominant	0.5625	0.2804–1.128	0.1053	1.18	0.7489–1.858	0.4761	0.77	0.3841–1.544	0.4614
	Recessive	1.38	0.5145–3.7	0.5226	0.6547	0.3174–1.351	0.2516	1.282	0.5792–2.836	0.5403
	Additive	0.8139	0.5011–1.322	0.4051	0.9997	0.7141–1.4	0.9987	0.9714	0.6229–1.515	0.8982
	Model-free	0.8113	0.4983–1.321	0.4001	0.9997	0.7049–1.418	0.9986	0.9703	0.6165–1.527	0.8961
*GGH* C452T (rs11545078)	Dominant	0.6364	0.293–1.382	0.2534	1.61	0.8131–3.186	0.1719	1.233	0.5681–2.676	0.5963
	Recessive	0.7073	0.04335–11.54	0.8079	NA^d^	NA	NA	NA	NA	NA
	Additive	0.6697	0.3287–1.365	0.2695	1.663	0.8667–3.19	0.1261	1.332	0.647–2.743	0.4365
	Model-free	0.6757	0.3352–1.362	0.2709	1.673	0.8709–3.213	0.119	1.327	0.6483–2.716	0.4378
*GGH* C401T (rs3758149)	Dominant	1.029	0.5249–2.016	0.9345	1.373	0.8587–2.194	0.1857	0.8942	0.4719–1.695	0.7317
	Recessive	0.5307	0.2047–1.376	0.1925	2.783	0.7604–10.19	0.122	1.429	0.4113–4.962	0.5745
	Additive	0.8653	0.5375–1.393	0.5514	1.412	0.945–2.111	0.09222	0.9916	0.5996–1.64	0.9737
	Model-free	0.8562	0.5231–1.401	0.5367	1.404	0.9442–2.087	0.09284	0.9915	0.5975–1.645	0.9736
*ATIC* C347G (rs2372536)	Dominant	0.4283	0.1974–0.929	0.03185	0.8524	0.541–1.343	0.4914	0.9651	0.481–1.936	0.9203
	Recessive	0.4542	0.1963–1.051	0.0652	1.523	0.7104–3.263	0.2798	0.6725	0.3155–1.434	0.3043
	Additive	0.5362	0.3258–0.8824	0.01421	0.9982	0.7116–1.4	0.9919	0.8615	0.553–1.342	0.5097
	Model-free	0.5478	0.3396–0.8835	0.01321	0.9982	0.7064–1.41	0.9917	0.8558	0.5444–1.345	0.4998
*ATIC* T675C (rs4673993)	Dominant	0.4504	0.2111–0.9611	0.03914	0.8334	0.5289–1.313	0.4322	0.8944	0.4428–1.806	0.7557
	Recessive	0.3682	0.1534–0.8834	0.02526	1.795	0.7934–4.06	0.1602	0.7808	0.3658–1.667	0.5225
	Additive	0.509	0.3072–0.8433	0.008759	1.008	0.7139–1.423	0.9642	0.8785	0.5627–1.371	0.5687
	Model-free	0.5247	0.3248–0.8478	0.008111	1.008	0.7119–1.427	0.9639	0.8748	0.5566–1.375	0.562
*ITPA* C94A (rs1127354)	Dominant	0.9471	0.4516–1.986	0.8855	1.04	0.6358–1.702	0.8749	0.9536	0.4676–1.945	0.896
	Recessive	0.3476	0.03078–3.925	0.3929	2.207	0.2269–21.46	0.4952	1.4	0.1242–15.77	0.7854
	Additive	0.879	0.4545–1.7	0.7015	1.075	0.678–1.705	0.7581	0.9863	0.5189–1.875	0.9665
	Model-free	0.8807	0.4578–1.694	0.7033	1.068	0.6883–1.657	0.7693	0.9866	0.5224–1.863	0.9668

^a^OR: odd ratio; ^b^95% CI: 95% confidence intervals frequency; ^c^*p*-value: probability value; ^d^No association analysis is possible.

**Table 6:** The asscoacition between SNPs and MTX toxicity in three different ancestry-specific groups.

SNP	Model	Malay			Chinese			Indian		
		OR^a^	95% CI^b^	p-value^c^	OR	95% CI	p-value	OR	95% CI	p-value
*FPGS* A1994G (rs10106)	Dominant	1.076	0.5332–2.173	0.8373	1.126	0.6935–1.828	0.6317	1.27	0.6375–2.528	0.4971
	Recessive	0.625	0.2149–1.817	0.3881	1.155	0.5432–2.456	0.7081	0.8569	0.3848–1.908	0.7053
	Additive	0.9271	0.5602–1.534	0.7684	1.1	0.7715–1.569	0.5975	1.054	0.6764–1.642	0.8167
	Model-free	0.929	0.5654–1.527	0.7714	1.108	0.7672–1.6	0.5845	1.056	0.6726–1.657	0.8137
*GGH* C452T (rs11545078)	Dominant	0.6417	0.2755–1.495	0.3038	1.261	0.6579–2.418	0.4846	1.513	0.7312–3.132	0.2642
	Recessive	1.063	0.09402–12.01	0.9609	NA^d^	NA	NA	7.855	0.8572–71.97	0.06821
	Additive	0.7077	0.3325–1.506	0.3698	1.163	0.6274–2.154	0.6321	1.667	0.9044–3.072	0.1014
	Model-free	0.7014	0.3271–1.504	0.3602	1.162	0.6279––2.15	0.6323	1.75	0.928–3.3	0.0812
*GGH* C401T (rs3758149)	Dominant	0.7945	0.402–1.57	0.5081	0.8385	0.5083–1.383	0.4903	0.8325	0.4405–1.573	0.5724
	Recessive	0.3116	0.08719–1.114	0.07274	0.8544	0.2683–2.721	0.7901	1.743	0.6346–4.787	0.2811
	Additive	0.6999	0.4225–1.159	0.1658	0.8658	0.5686–1.318	0.5019	1.017	0.6335–1.632	0.945
	Model-free	0.6805	0.4042–1.146	0.1464	0.8657	0.5685–1.318	0.5011	1.018	0.6206–1.671	0.9424
*ATIC* C347G (rs2372536)	Dominant	1.014	0.4782–2.148	0.9721	1.006	0.6201–1.633	0.9794	0.6387	0.3253–1.254	0.1927
	Recessive	0.945	0.3951–2.261	0.8988	1.4	0.6652–2.947	0.3754	1.348	0.637–2.853	0.4349
	Additive	0.9875	0.6009–1.623	0.9604	1.082	0.7558–1.548	0.6674	0.9168	0.5907–1.423	0.6984
	Model-free	0.9882	0.6098–1.601	0.9615	1.086	0.7522–1.568	0.6598	0.9127	0.5817–1.432	0.691
*ATIC* T675C (rs4673993)	Dominant	1.111	0.5266–2.344	0.7821	0.9631	0.5935–1.563	0.879	0.6097	0.3097–1.2	0.1522
	Recessive	0.8729	0.3527–2.16	0.7688	1.401	0.6466–3.036	0.3926	1.23	0.575–2.632	0.5933
	Additive	1.006	0.61–1.657	0.9827	1.052	0.7298–1.517	0.7857	0.8689	0.5565–1.357	0.5366
	Model-free	1.005	0.6195–1.631	0.9833	1.053	0.7276–1.525	0.7833	0.8657	0.5514–1.359	0.5307
*ITPA* C94A (rs1127354)	Dominant	1.034	0.4869–2.198	0.9297	1.08	0.6414–1.82	0.7713	0.8193	0.3939–1.704	0.5937
	Recessive	4.383	0.3878–49.54	0.2324	NA	NA	NA	3.789	0.3364–42.69	0.281
	Additive	1.162	0.5949–2.27	0.6603	0.9981	0.6101–1.633	0.9939	0.9455	0.4914–1.819	0.8667
	Model-free	1.158	0.5979–2.242	0.6638	0.9983	0.6255–1.593	0.9942	0.9462	0.4941–1.812	0.8676

^a^OR: odd ratio; ^b^95% CI: 95% confidence intervals frequency; ^c^*p*-value: probability value; ^d^No association analysis is possible.

## Discussion

Majority of the available drugs used for the treatment of RA were clinically evaluated in European ancestries, this raises a concern about their efficacy and toxicity in other ancestry groups globally^32^. Asian populations especially those in South East Asia were considerably under-represented in pharmacogenomic and pharmacogenetic studies of RA^32,33^. Hence, the present study evaluated the outcomes of MTX treatment in three major ancestry groups in Malaysia and their association with 6 SNPs from the enzymes involved in MTX metabolism. Comparing with the studies conducted by geographical locations, our study attempted to delineate ancestry specific risk factors that would increase the precision of the proposed association.

The MAF of *ATIC* T675C (rs4673993) recorded in Genome Aggregation Database (gnomAD) and 1000 Genomes is 0.3251 and 0.2855, respectively^34,35^. In our study, the allele frequency of *ATIC* T675C (rs4673993) for the overall cohort is 0.4 and it is 0.44, 0.31 and 0.54, respectively, in Malay, Chinese and Indian populations in Malaysia (Table 2). By comparing the allele frequency between our study and the public database, we noticed that our population is carrying a higher allele frequency of *ATIC* T675C (rs4673993). When comparing allele frequencies by ethnicity within our study cohort, there was a significant difference between Malay, Chinses and Indian for this SNP. Apparently, the allele frequency of *ATIC* T675C (rs4673993) in Indian and Malay subjects were significantly higher than that observed in Chinese subject and in the public databases.

Interestingly, our study suggested that the Malay RA patients with minor allele of *ATIC* T675C (rs4673993) have a better treatment outcome upon MTX monotherapy. In other words, this minor allele was associated with an increased remission rate in Malay RA patients following the treatment of MTX. A few studies have also demonstrated the impact of *ATIC* T675C (rs4673993) on MTX treatment outcome. Prospective studies conducted by Lee et al. and Iannaccone et al. have shown that the minor allele of *ATIC* T675C (rs4673993) was significantly associated with low disease activity in RA patients with MTX monotherapy^23,24^. These two studies were conducted in the USA with 120 and 262 RA patients, respectively, as the subsets of Brigham and Women’s Hospital Rheumatoid Arthritis Sequential Study (BRASS). Moreover, a meta-analysis performed by Chen (2017) indicated that *ATIC* T675C (rs4673993) could predict the responsiveness of MTX treatment in recessive model (OR 2.54; 95% CI 1.23–5.26)^36^. The authors combined two studies to yield a total of 698 Caucasians and observed a significant favouritism of *ATIC* T675C (rs4673993) in RA patients having response to MTX treatment. On the other hand, a retrospective study by Lima et al. (2014) gave a totally different conclusion^37^, whereby more than fourfold increase in risk of MTX inefficacy was associated with *ATIC* T675C (rs4673993) in a population of 233 adults ($\ge$ 18 y.o.) of Portuguese Caucasian RA patients. The result discrepancy may be due to different ancestral lineages of RA patients enrolled in the respective studies. In our case, the association of polymorphism *ATIC* T675C (rs4673993) with the responsiveness to MTX treatment could only be observed in Malay but not in Chinese and Indian RA patients.

Current RA literature consistently highlights the hypothesis that the anti-inflammatory action of MTX is achieved through the indirect inhibition of AICAR. The *ATIC* T675C (rs4673993) SNP is positioned in the intronic region of *ATIC*. To our knowledge, there are no functional studies on this particular SNP in ATIC activity. Nevertheless, the intronic SNP either interferes the transcriptional regulation of the coding-enzyme or is in linkage disequilibrium (LD) with another coding SNP^23,38–40^. In the present study, since similar effect size of *ATIC* T675C (rs4673993) and *ATIC* C347G (rs2372536) was observed, both SNPs can be in LD. Nevertheless, this observation need further validation, since the current sample size is too small to perform a LD test and the lack of a reference panel for *ATIC* T675C (rs4673993) and *ATIC* C347G (rs2372536) in Malay patients.

Similar to *ATIC* T675C (rs4673993), the allele frequency of *ATIC* C347G (rs2372536) in Malay, Chinese and Indian populations is 0.45, 0.31 and 0.54, respectively; and the allele frequency of *ATIC* C347G (rs2372536) for the entire study cohort is 0.40 (Table 2). The allele frequency of *ATIC* C347G (rs2372536) retrieved from gnomAD, GO-ESP and 1000 Genomes are 0.3172, 0.2468 and 0.2778^34,35,41^, respectively. Except the Chinese population, the allele frequencies observed for Malay and Indian are higher than the ones retrieved from the public databases.

Our result suggested Malay RA patients with minor allele G of *ATIC* (rs2372536) showing a better response to MTX treatment as compared to Chinese and Indian RA patients. In fact, this is in alignment with the data previously presented by Dervieux et al.^38^ on a cross-sectional study of 108 RA patients (age ≥ 18 y.o.) from a local rheumatology clinic in Knoxville, USA. In their study, patients carrying a homozygous GG of *ATIC* C347G (rs2372536) may have a higher ratio of good response to MTX compared with patients carrying a CC or CG genotype. Moreover, Kurzawski et al.^22^ studied 422 Caucasian RA patients in Poland who were treated with MTX therapy and found that GG minor genotype significantly exhibited a good response to MTX. However, the lack of association between rs2372536 polymorphism and the clinical response to MTX was also reported in some studies^42,43^. Recently, two meta‐analyses were performed to investigate the association between *ATIC* C347G (rs2372536) and MTX response^44,45^. The first meta‐analysis was based on five studies of 1056 RA patients in which 722 were MTX responders and 334 were non-responders. This analysis found the difference of *ATIC* C347G (rs2372536) between Caucasions (Spain, Slovenia and Netherlands) and Asians (India), being associated with non-responsiveness to MTX treatment in Caucasians but not associated in Asians^44^. The second meta‐analysis combined two European (Spain and Netherlands), one East Asian (Japan) and two South Asian (India) studies with 458 MTX responders and 398 non-responders in total^45^. When combining five studies, *ATIC* C347G (rs2372536) demonstrated a significant association with non-responsiveness of MTX under the dominant and codominant models. Yet, geographical stratification showed that the association of *ATIC* C347G with MTX response was still observed in Europeans in pre-allele, dominant and codominant models but not in South Asian populations^44^.

Despite all studies above demonstrated a significant association between *ATIC* variants and MTX efficacy, the results were raher inconsistent. Common factors for inconsistency such as small sample size and insufficient statistical power, study design, medication dosage, grouping criteria, and patient condition could cause limitations in the association study. Moreover, gene–gene interactions within folate and adenosine biosynthesis pathways may complicate the association study between SNPs and MTX treatment outcomes^46^. In fact, RA has complex inheritance patterns and no single genetic variant has a decisive role in MTX efficacy or MTX toxicity in the treatment of RA. By using the MDR (Multifactor Dimensionality Reduction) method, a cohort of 255 RA patients treated with MTX in the USA was evaluated with the efficacy of MTX treatment, and the results showed that 53% MTX responders was associated with high-order interactions among SNPs in *ITPA* (C94A), *RFC*1 (G80A), and *ATIC* (C347G) genes^46^. Upon excluding the predisposing genotype combinations, a 3.8-fold lower efficacy was observed^46^. Later, the same researchers extended their study of gene–gene interactions using *ITPA* (C94A), *RFC*1 (G80A), and *ATIC* (C347G) to another 3 RA cohorts (USA, Dutch and Swedish)^47^. Both USA and Dutch cohorts (n = 435) confirmed a predisposing genetic attribute significantly associated with methotrexate response [odds ratio (OR) = 2.9, 95% confidence interval (CI) 1.9–4.2; *P* < 0.001]. Although the association of combined SNPs with MTX responsiveness in the Swedish cohort (n = 530) could not be determined, the association was observed after the non-genetic factors, age, sex and anti-citrullinated protein antibody (ACPA) status were included in MDR analysis^47^. Thus, individual variants of *ATIC* may not play a direct role in MTX efficacy, future studies shall map the *ATIC* variants to drug response as based on the detection of nonlinear multigene interactions, this may improve the accuracy of predicting the MTX efficacy. In addition, other non-genetic covariates should be considered because the association study between genetic variants and MTX efficacy sometimes seems oversimplified understanding the MTX response in RA.

AICAR transformylase contains two domains which are MGS (methylglyoxal synthetase) like domain and AICAR binding domain^48^. *ATIC* C347G (rs2372536) causes the substitution of threonine (Thr) with serine (Ser) at position 116. Thr116 lies in the binding pocket of MGS-like domain and is the first residue of α8 helix which likely serves as a N-cap residue stabilizing the helix by interacting with the amide groups from the main chain. We proposed that the side-chain hydroxyl group of Thr116 forms hydrogen bonds with the amide groups of Val117 and Glu118 (green arrowhead in Supplementary Fig. S2), while its main-chain carboxyl group forms hydrogen bond with the amide group from Glu119 (blue arrowhead in Supplementary Fig. S2). The methyl group of Thr116 might stabilize the hydrogen bond between Thr116 and the main chain. As Thr116 is substituted with serine, the methyl group can be removed and this results in a more flexible C-N rotation. In other words, Ser116 causes the rearrangement of the protein structure at N-cap and thus, potentially affects the substrate-binding affinity and AICAR transformylase enzyme activity. This explains why the RA patients with the minor allele of *ATIC* C347G (rs2372536) might have a phenotypic change in response to MTX.

## Conclusion

Present study suggested that minor allele of *ATIC* C347G (rs2372536) and *ATIC* T675C (rs4673993) could influence the response to MTX monotherapy in Malay patients with RA, while the other four SNPs failed to demonstrate their associations with the reduction of disease activity following the MTX monotherapy. *ATIC* C347G (rs2372536) and *ATIC* T675C (rs4673993) are not the only ancestry-specific SNPs since any variations appear in the genes of MTX metabolic pathway are potentially able to affect the effectiveness of MTX treatment. As more Malay-specific SNPs can be revealed, the prediction of poor response would enable patient to be placed on alternative drugs, while those with predicted good response could proceed with MTX treatment.

As for the future recommendation, ancestry specific signal of *ATIC* should be validated in a larger replication cohort of a similar ancestry group profile to reduce the Type II error rate of MTX treatment response. Both *ATIC* C347G (rs2372536) and *ATIC* T675C (rs4673993) warrant an in-depth investigation, especially in the Malay RA patients in Malaysia. Having said that, other gene variants affecting MTX efficacy and toxicity should not be neglected, they may have potential impact on Malaysian RA patients treated with MTX. Among them, SLC19A1/RFC1, ABCB1 and MTHFR variants are well documented in many studies. RFC1 (reduced folate carrier), which is also known as SLC19A1, transports MTX into the cell while ABCB1 (ATP Binding Cassette B1 or P-glycoprotein) is ATP-dependent pump that moves MTX out of the cell. Gene variants of SLC19A1/RFC-1 (rs1051266 and rs2838956) and ABCB1 (rs1045642 and rs1128503) have been shown to influence MTX influx and efflux in and out of the cell, respectively, leading to the therapeutic effects^49–52^. MTHFR (methylenetetrahydrofolate reductase) is another well studied metabolic gene of MTX and it has been associated with the MTX inadequate responders. Nucleotide substitutions in MTHFR such as C677T (rs1801133) and A1298C (rs1801131) which result in single amino acid substitutions can greatly reduce the production of functional reductase and its enzymatic activity^53^. Last but not least, any variations in enzymes involved in the intracellular MTX metabolic pathway may affect functional properties of the enzyme and hence the responses to MTX efficacy and toxicity.

## Methods

### Study subjects

RA patients were recruited at Sunway Medical Centre (Selangor, Malaysia), Hospital Tuanku Ja’afar Seremban (Seremban, Malaysia) and Hospital Selayang (Selangor, Malaysia) from December 2016 to May 2019. The study was performed in accordance with the principles stated in the Declaration of Helsinki. Prior to starting the study, the ethical approval was obtained from the Sunway University Research Ethics Committee (SUNREC 2017/066), the Sunway Medical Centre Independent Ethics Committee (007/2016/ER), and the Medical Research Ethics Committee of Ministry of Health Malaysia (NMRR-17-2901-38245(IIR). RA patients enrolled in this study had fulfilled ACR-EULAR (2010) response criteria and satisfy the inclusion criteria: (i) must be at least 18 years old, (ii) are Malaysian Malay, Chinese or Indian origin, (iii) have been treated with 15 mg MTX or more per week for at least 3 months and (iv) have been followed up 6 months since MTX treatment. The self-declared ancestry of a patient was decided based on both parents being of the same ancestry as the patient. Patients of non-Malaysian origin were excluded from this study. All recruited RA patients were subsequently subjected to two independent association studies on the potential pharmacokinetic impact of SNPs with MTX efficacy and with MTX toxicity.

#### MTX efficacy

RA patients were DMARD naive at the time of MTX commencement and they were categorized into adequate responder (AR) and inadequate responder (IR). Adequate responders were interpreted as patients who are in clinical remission or have achieved low disease activity as defined by Disease Activity Score-28 (DAS28CRP) for at least 6 months. On the other hand, inadequate responders have the same RA treatment as adequate responders but failed to achieve clinical remission or low disease activity as defined by DAS28CRP but at present been treated with other DMARDs, mono, duo (excluding MTX and HCQ group) or triple therapy, either csDMARDs or tsDMARDs or bDMARDs. Hence, patients may or may not be on MTX at the point of recruitment.

#### MTX toxicity

Patients were categorized into two groups for potential toxicity and side effect: non-adverse drug reaction (Non-ADR) group and adverse drug reaction (ADR) group. The categorization was marked based on whether they have experienced drug intolerance during the MTX treatment. All side effects were recorded from the start of the MTX treatment until the withdrawal due to adverse drug reactions.

### Blood sample and clinical data collections

A total of 647 RA patients were involved in this study after their informed consent was obtained. A total of 5 ml of the whole blood sample was collected in ethylenediaminetetraacetic acid (EDTA) tube from individual patients by venepuncture during patients’ regular visits at the hospitals. Besides, clinicopathological and demographic data were also extracted from patients’ clinical records and linked to deidentified patient blood samples collected for this study.

### TaqMan^®^ SNP genotyping assays

Genomic DNA from patients was isolated from patients’ whole blood samples. Briefly, buffy coat was obtained from the blood sample by centrifugation. Genomic DNA was then extracted from the buffy coat by using QIAamp DNA Blood Mini Kit (Qiagen, Germany) according to the manufacturer’s instructions. SNP genotyping of *FPGS* A1994G (rs10106), *GGH* C452T (rs11545078), *GGH* C401T (rs3758149), *ATIC* C347G (rs2372536), *ATIC* T675C (rs4673993) and *ITPA* C94A (rs1127354) were performed by using TaqMan® SNP Genotyping Assays (Thermo Fisher, USA) according to manufacturer’s instructions. The genotype data of each participant were analyzed using an online software named “Genotyping V4.2” (Thermo Fisher Connect™). A total of 5% of the samples (n = 33) for each respective SNP were randomly selected for PCR amplification (AmpliTaq Gold™ 360 Master Mix, Thermo Fisher Scientific, USA) (Supplementary Table S1) and subsequently for the genotype verification by Sanger sequencing (1st BASE Pt Ltd). Sequencing results were curated with SnapGene V4.3.10 (from GSL Biotech; available at https://snapgene.com).

### Statistical analysis

Genotype and allele frequency of all the selected six SNPs were calculated. A chi-square independence test was performed to test the association between SNPs and ethnic groups. Chi-square test and binary logistic regression were performed to investigate the association between SNPs and MTX efficacy and MTX toxicity (PLINK V1.09)^54^. Effect sizes of potential associations were calculated as odds ratio (OR) and 95% confidence intervals (CI) as a measure of the association between the categorical variables. A p-value of < 0.05 was considered to be statistically significant.

## Supplementary Information

Supplementary Information.

## Data Availability

The data underlying this article cannot be shared publicly due to the privacy of individuals that participated in the study. The data will be shared on reasonable request to the corresponding author.
