# Supplementary material for: Genetic variations in methotrexate metabolic pathway genes influence methotrexate responses in rheumatoid arthritis patients in Malaysia
Source: Sci Rep. 2022 Jul 13;12:11844. doi: 10.1038/s41598-022-15991-0 (PMC9279481; doi:10.1038/s41598-022-15991-0)
Supplement: Supplementary file 1 — Supplementary Information. [file 41598_2022_15991_MOESM1_ESM.pdf]

Supplementary Table S1 Verification primers for amplification of target region containing specific SNP

Primer Name	Sequence 5’-> 3’	Tm (ºC)	Product size (bp)
rs11545078-FW	CATGTTTTCCAGCCTGTGTG	54.5	508
rs11545078-RV	GGATGGTCATTCACATCTTCAA	52.9	
rs1127354-FW	TAGGGTTTCTGGGAGCATTG	54.6	548
rs1127354-RV	CCTGGAAGCTACCTGGACAA	56.6	
rs10106-FW	AGGAAATCTACCACCCAGCA	55.9	580
rs10106-RV	CGTCTTCAGCTGCATTTCAC	54.5	
rs2372536-FW	CAGCAGGACACCCTGACTTT	57.3	590
rs2372536-RV	AGGTTGCAGTCAGCCAAAAC	56.3	
rs4673993-FW	GCTGTCCGATGGGTCTGTAT	56.6	588
rs4673993-RV	CCAGCCTGCAGTATTGTGTC	56.1	
rs3758149-FW	GTGCCCGCTTCAAAGTAGAC	56.2	594
rs3758149-RV	GGCCTATGGGAAATGCAGTC	56.1	

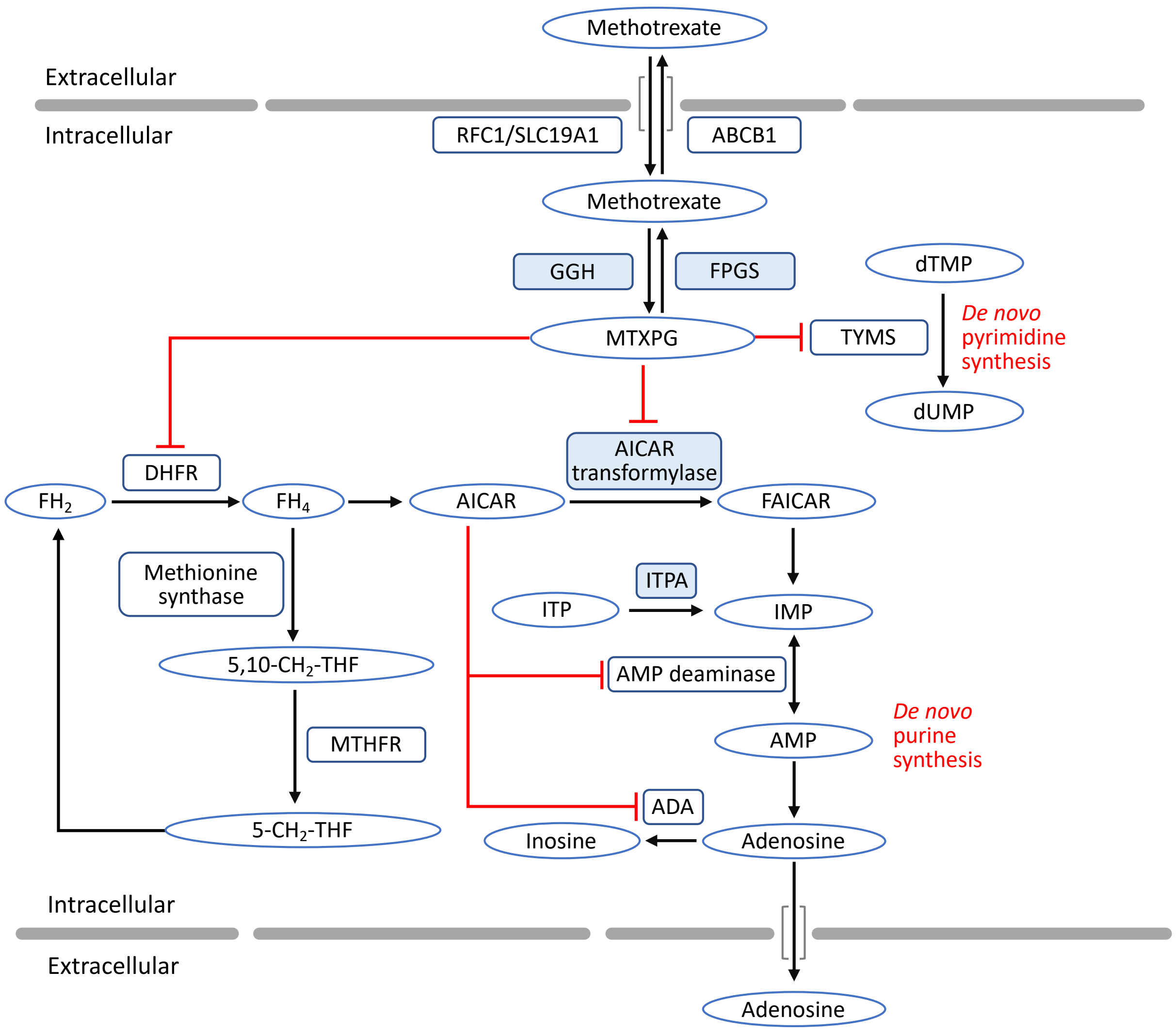

Supplementary Fig. S1. Cellular pathway of MTX — uptake, transport, conversion to polyglutamate forms and downstream effects. MTX is absorbed through active transport mediated by reduced folate carrier (RFC1) or solute carrier family 19 member 1 (SLC19A1). Reversely, MTX is pumped out from the cell via the ATP Binding Cassette B1 (ABCB1). Inside the cell, MTX is converted to active methotrexate polyglutamates (MTX PGs) by folylpolyglutamate synthase (FPGS) and this process can be reversed by γ-glutamyl hydrolase (GGH). MTX PGs directly inhibit dihydrofolate reductase (DHFR), aminoimidazole-4-carboxamide ribonucleotide (AICAR) transformylase which is coded by *ATIC* and thymidylate synthetase (TYMS). Proteins highlighted in blue are encoded by the genes chosen for genotyping in this study. Red diamond (◆) indicates the inhibitory activity and black arrowhead (▼) indicates the directional flow of reaction.

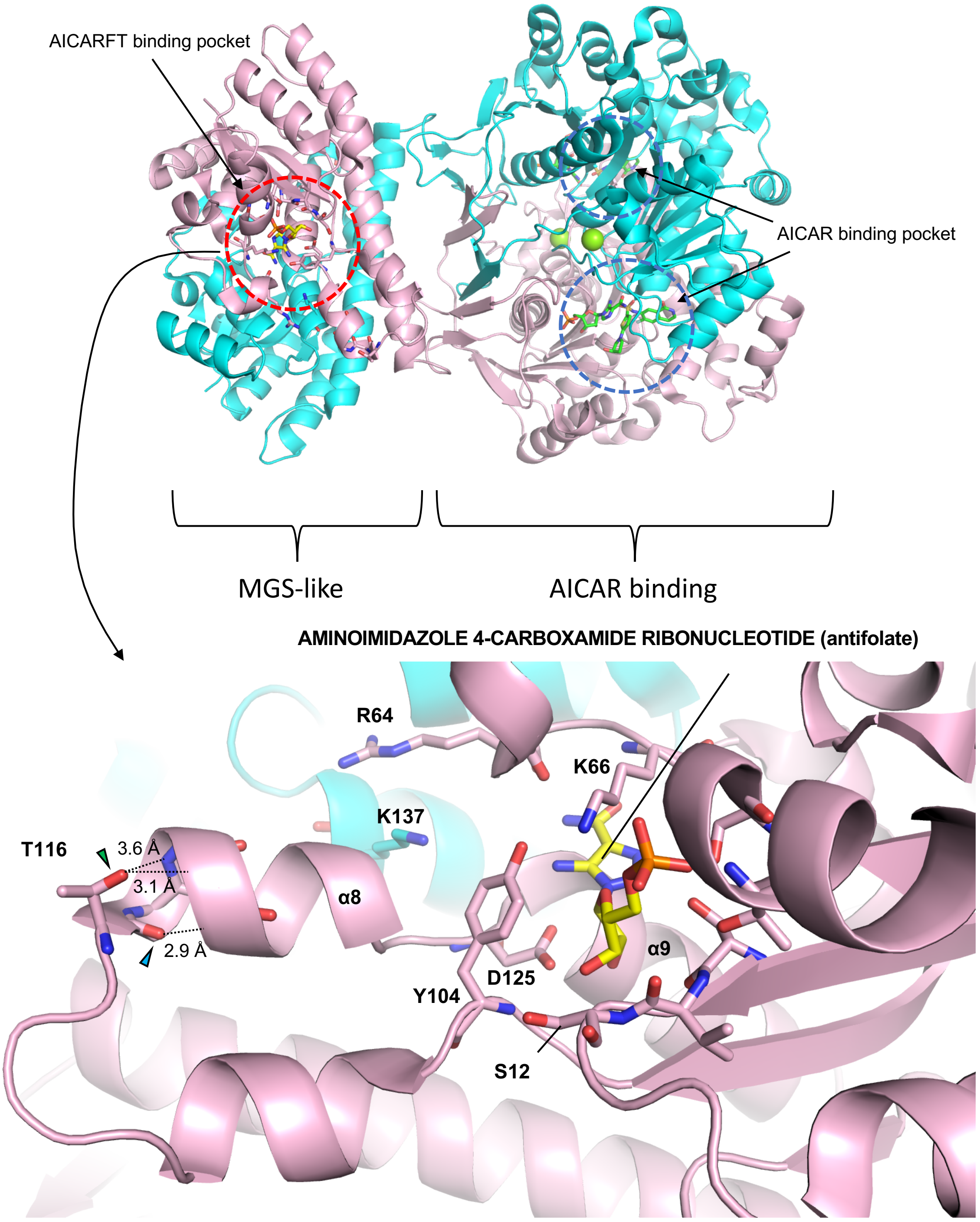

Supplementary Fig. S2. Tertiary structure of AICAR transformylase complexed with antifolate (aminoimidazole 4-carboxamide ribonucleotide) was retrieved from Protein Data Bank (https://www.rcsb.org) (PDB ID: 5UZ0)[49]. Thr116 (or T116) is in the MGS domain. The green arrowhead indicates the side-chain hydroxyl group of Thr116 that forms hydrogen bonds with the amide group from Val117 and Glu118. The blue arrowhead is where the main-chain carboxyl group of Thr116 forms hydrogen bond with the amide group of Glu119.
